# Supplementary material for: Design, Synthesis and Biological Evaluation of Novel N-Arylpiperazines Containing a 4,5-Dihydrothiazole Ring
Source: Pharmaceuticals (Basel). 2023 Oct 18;16(10):1483. doi: 10.3390/ph16101483 (PMC10609883; doi:10.3390/ph16101483)
Supplement: Supplementary file 1 [file pharmaceuticals-16-01483-s001.zip › pharmaceuticals-2635195-supplementary.pdf]

Supporting Information

Design, synthesis, and biological evaluation of novel N-arylpiperazines containing a 4,5 dihydrothiazole ring.

Giorgia Andreozzi[1], Maria Rosaria Ambrosio[2], Elisa Magli[1], Giovanni Maneli[4], Beatrice Severino[1], Angela Corvino[1], Rosa Sparaco[1], Elisa Perissutti[1], Francesco Frecentese[1], Vincenzo Santagada[1], Anna Lesniak[5], Magdalena Bujalska-Zadrozny[5], Giuseppe Caliendo[1], Pietro Formisano[2,4] and Ferdinando Fiorino[1]*

[1]Dipartimento di Farmacia Università di Napoli Federico II Via D. Montesano, 49, 80131, Naples, Italy

[2]URT "Genomic of Diabetes", Institute for Experimental Endocrinology and Oncology "G. Salvatore", National Research Council (IEOS-CNR), Via Pansini 5, 80131 Naples, Italy

[3]Dipartimento di Sanità Pubblica Università di Napoli Federico II Via Pansini, 5, 80131, Naples, Italy

[4]Department of Translational Medicine, University of Naples "Federico II", Via Pansini 5, 80131 Naples, Italy.

[5]Department of Pharmacotherapy and Pharmaceutical Care, Centre for Preclinical Research and Technology, Faculty of Pharmacy, Medical University of Warsaw, 1 Banacha Str., 02-097 Warsaw, Poland

*Correspondence: Corresponding author. Tel.: +39-81 679825; fax 39-81-678649; e-mail address: fefiorin@unina.it

Contents: ^{1}H and ^{13}C NMR spectra for 2a-2c, 3a-3c

Compound 2a

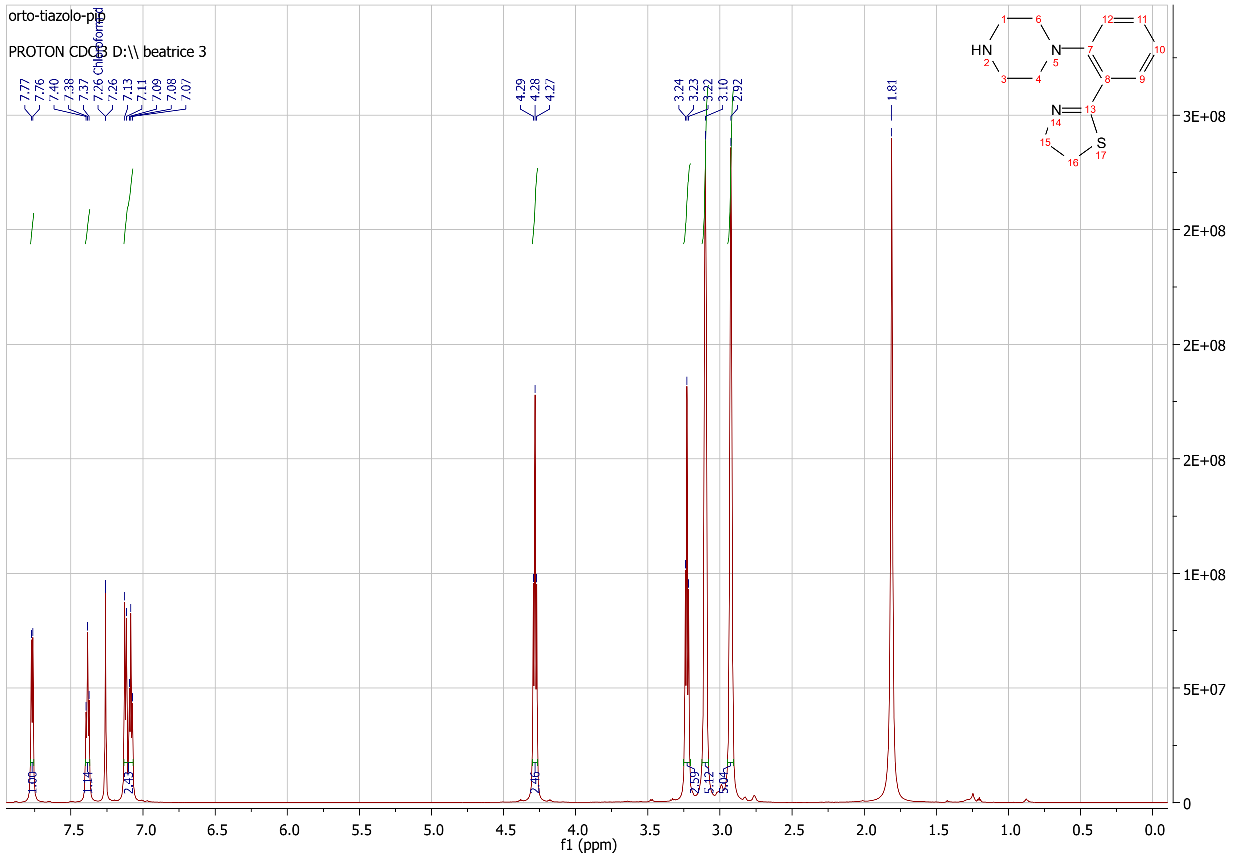

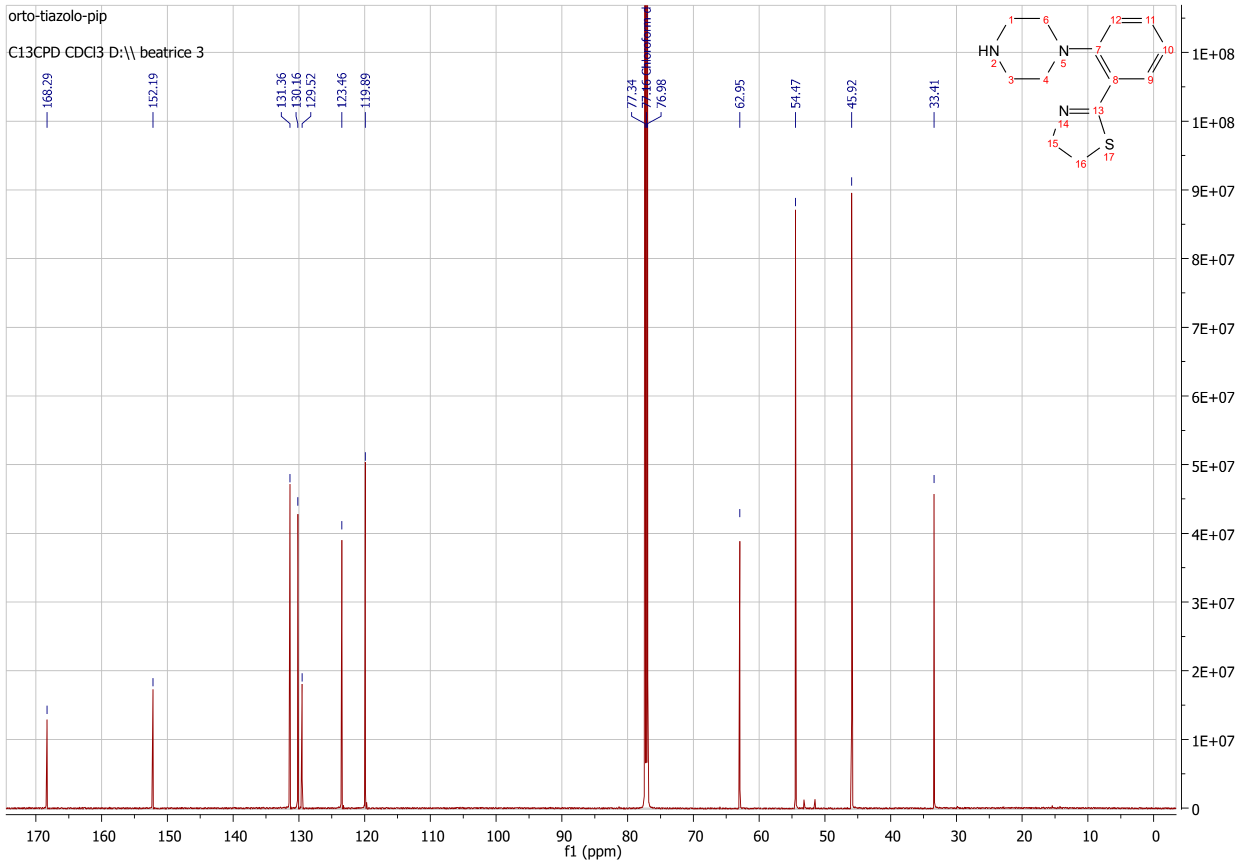

Compound 2b

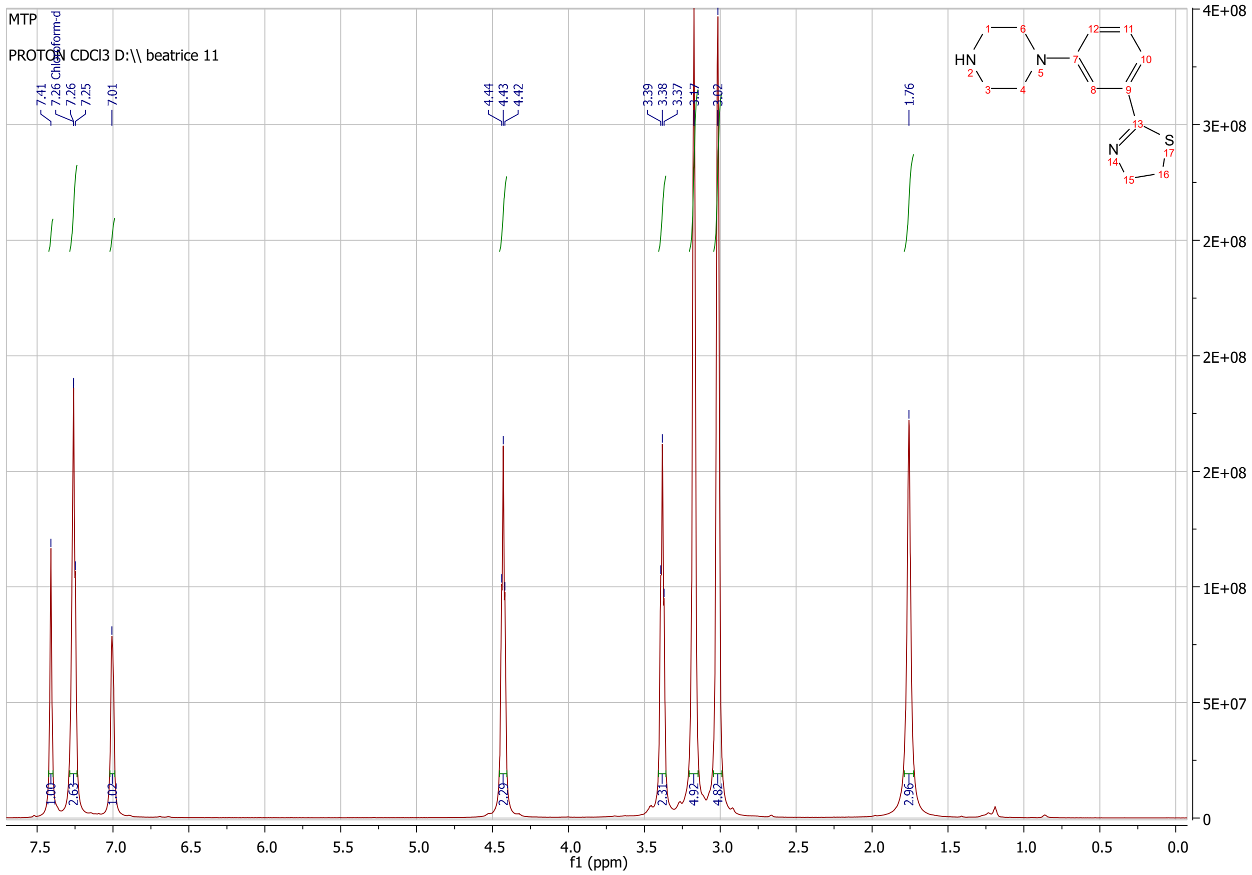
MTP
PROTON CDCl3 D:\\ beatrice 11
7.41
7.26 Chloroform-d
7.26
7.25
7.01
4.44
4.43
4.42
3.39
3.38
3.37
3.17
3.02
1.76
1.00
2.63
1.02
2.29
2.31
4.92
4.82
2.96
f1 (ppm)
HN
N
S
N

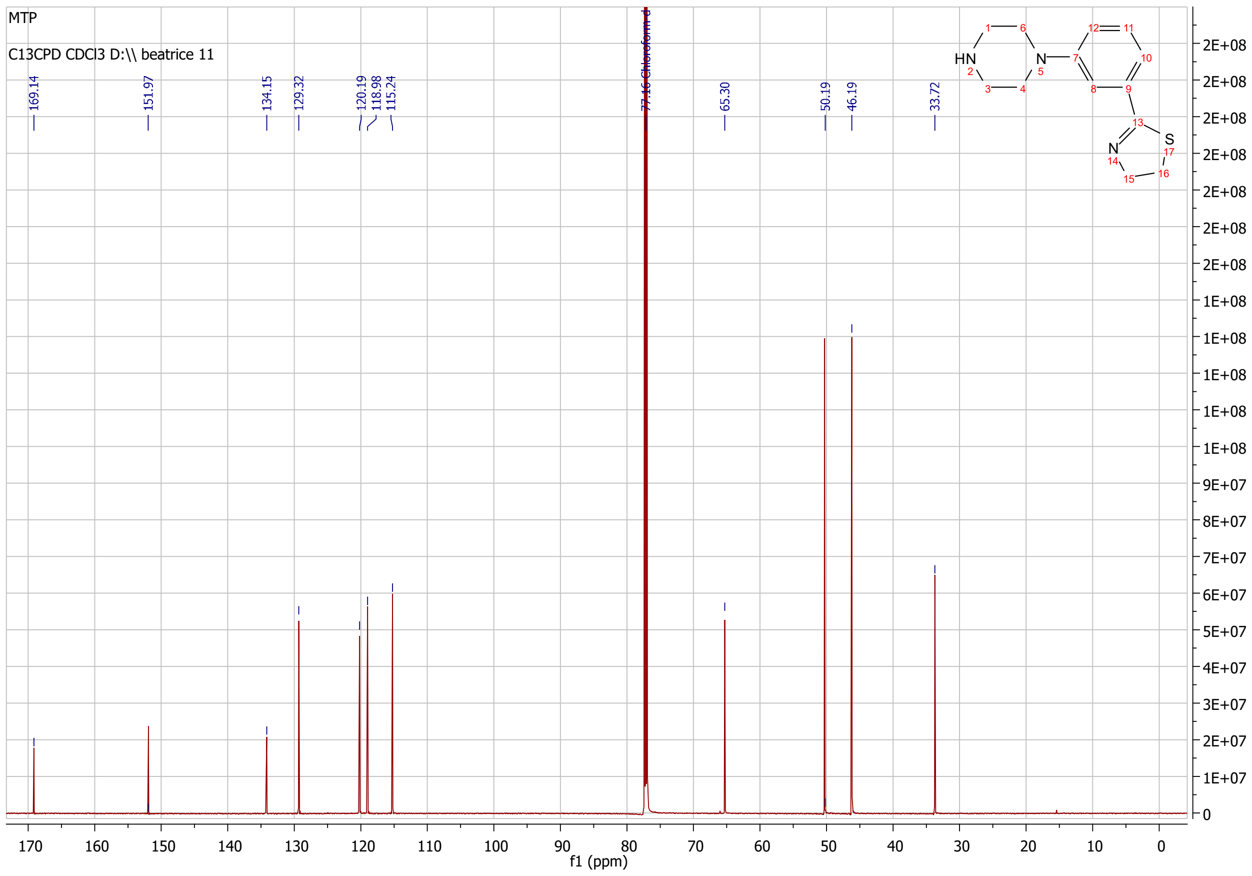
MTP
C13CPD CDCl3 D:\\ beatrice 11
169.14
151.97
134.15
129.32
120.19
118.98
115.24
77.16 Chloroform-d
65.30
50.19
46.19
33.72
f1 (ppm)
HN
N
S
N

Compound 2c

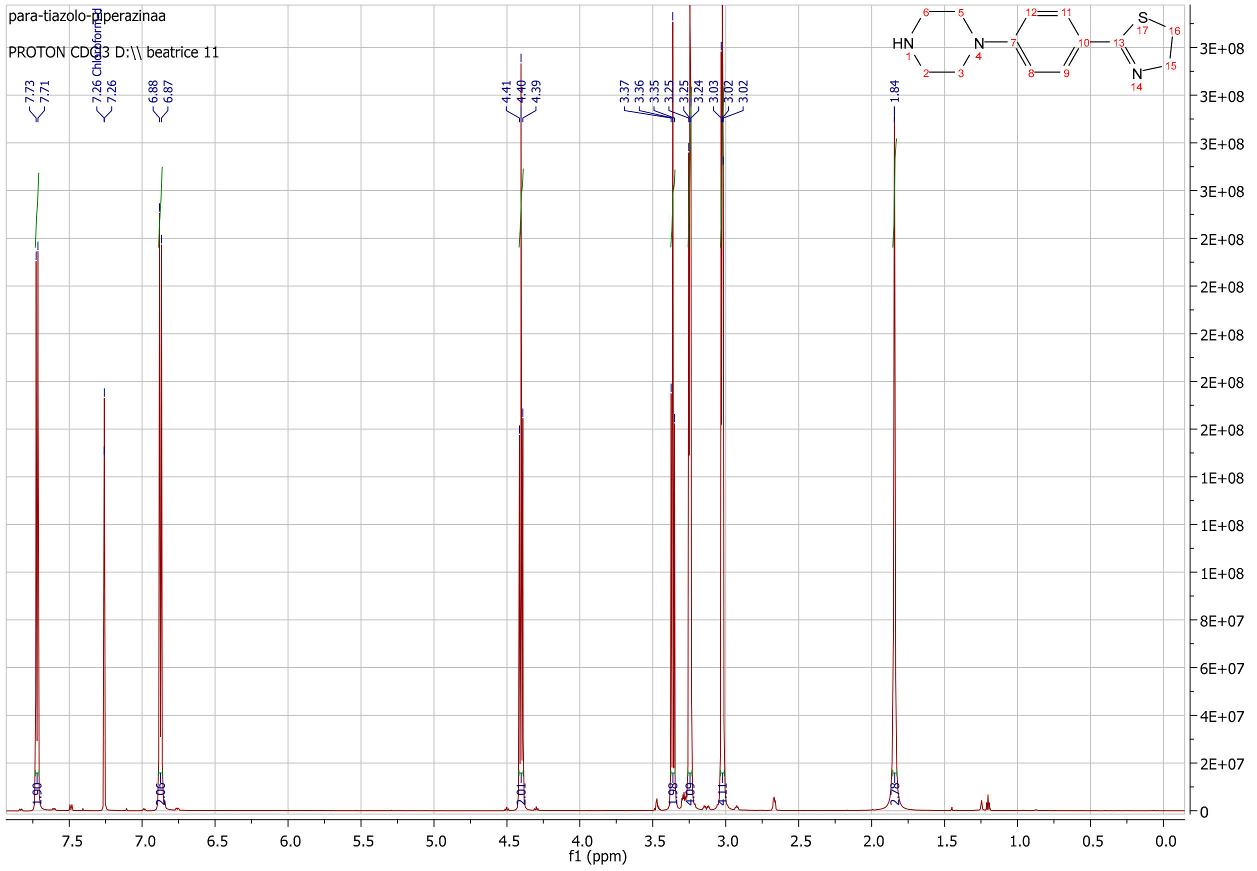
para-tiazolo-piperazinaa
PROTON CDCl3 D:\\ beatrice 11
7.73
7.71
7.26 Chloroform-d
7.26
6.88
6.87
4.41
4.40
4.39
3.37
3.36
3.35
3.25
3.24
3.03
3.02
3.02
1.84
2.06
2.01
4.09
4.11
2.78
f1 (ppm)
HN
N
S
N

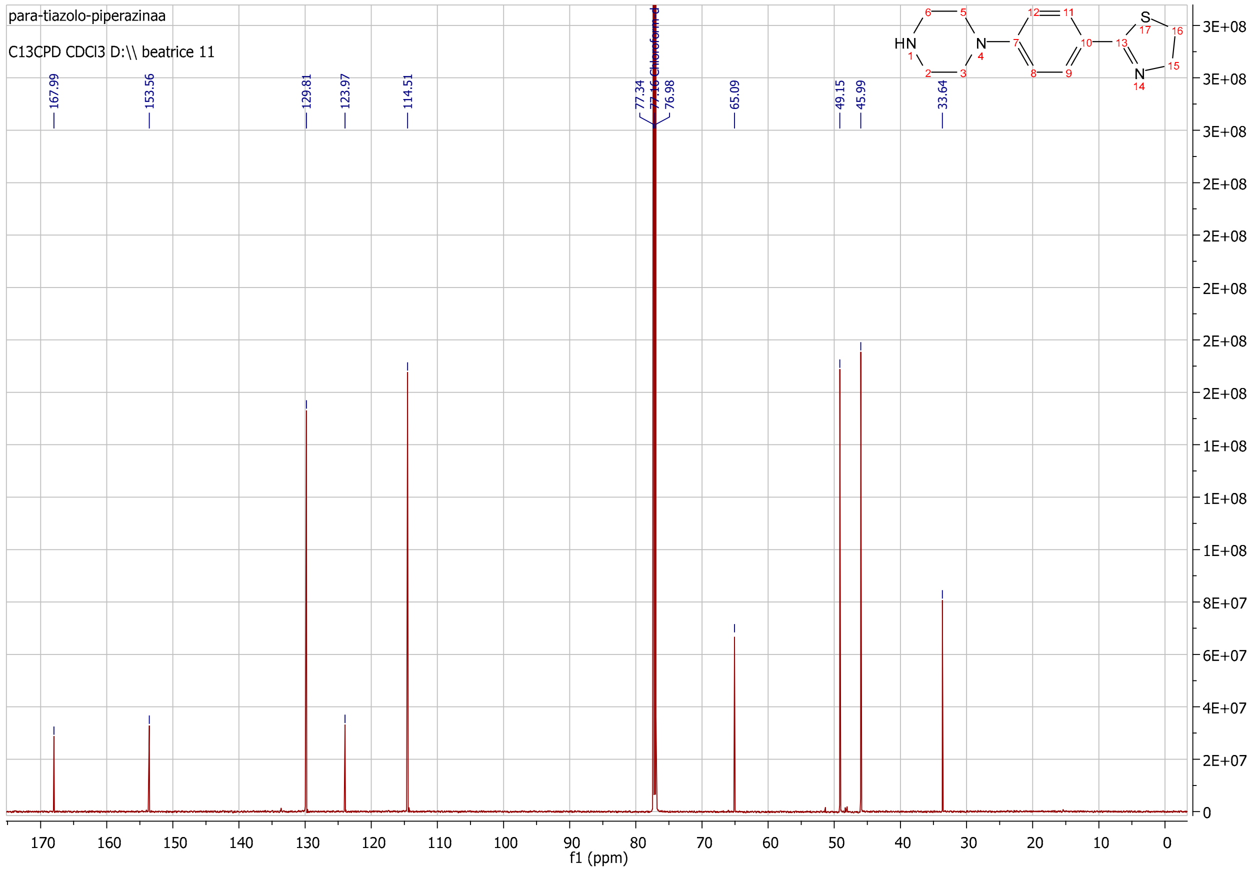
para-tiazolo-piperazinaa
C13CPD CDCl3 D:\\ beatrice 11
167.99
153.56
129.81
123.97
114.51
77.34
77.16 Chloroform-d
76.98
65.09
49.15
45.99
33.64
f1 (ppm)
HN
N
S
N

Compound 3a

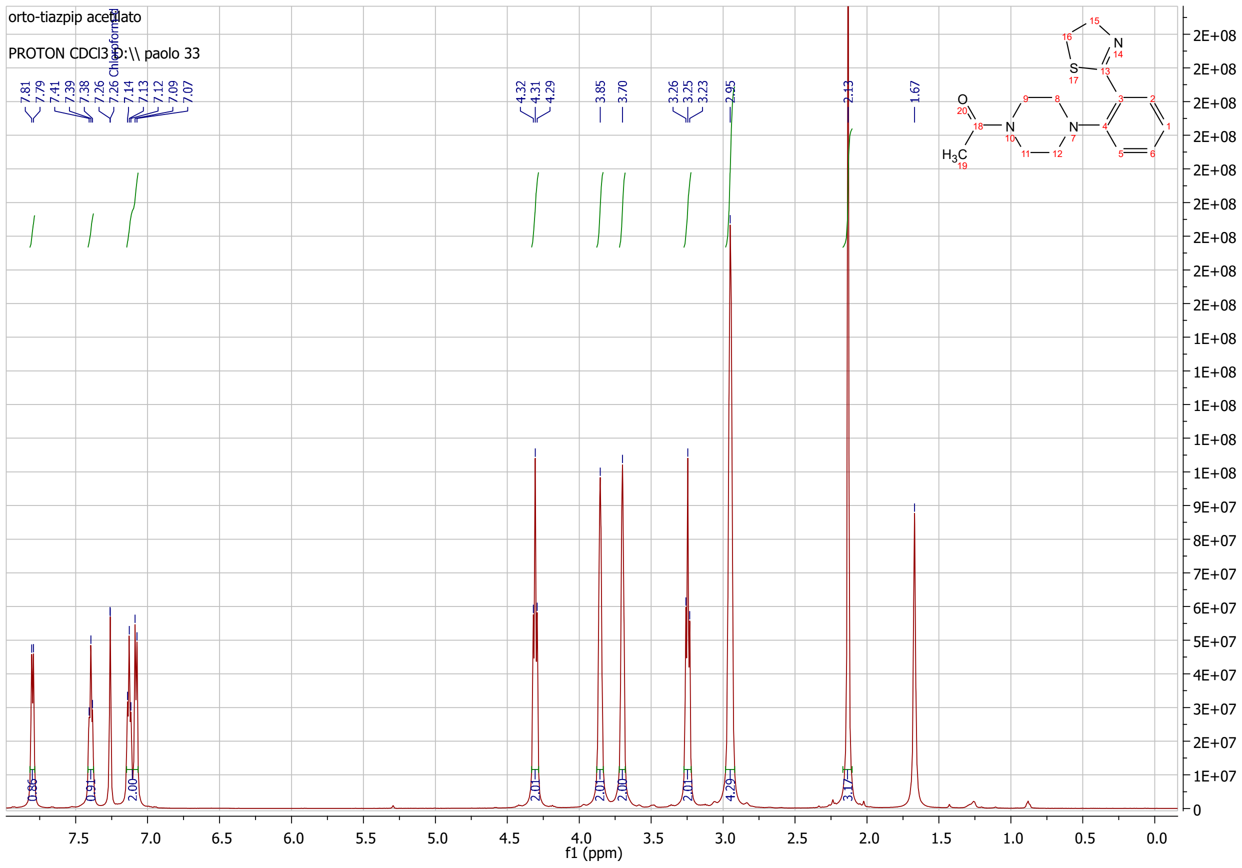

orto-tiazpip acetilato
PROTON CDCl3 D:\\ paolo 33
7.81
7.79
7.41
7.39
7.38
7.26
7.26 Chloroform-d
7.14
7.13
7.12
7.09
7.07
4.32
4.31
4.29
3.85
3.70
3.26
3.25
3.23
2.95
2.13
1.67
0.86
0.91
2.00
2.01
2.01
2.00
2.01
4.29
3.17
f1 (ppm)

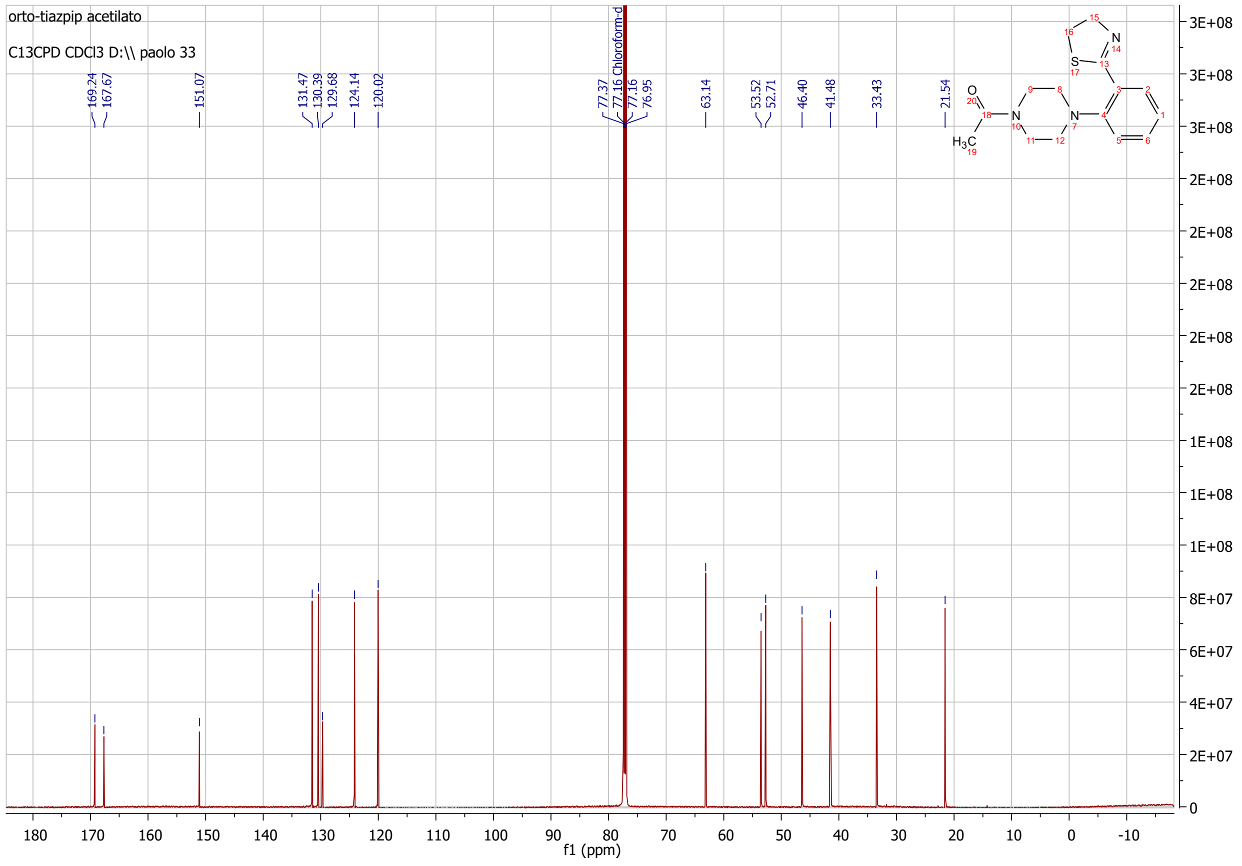

orto-tiazpip acetilato
C13CPD CDCl3 D:\\ paolo 33
169.24
167.67
151.07
131.47
130.39
129.68
124.14
120.02
77.37
77.16 Chloroform-d
77.16
76.95
63.14
53.52
52.71
46.40
41.48
33.43
21.54
f1 (ppm)

Compound 3b

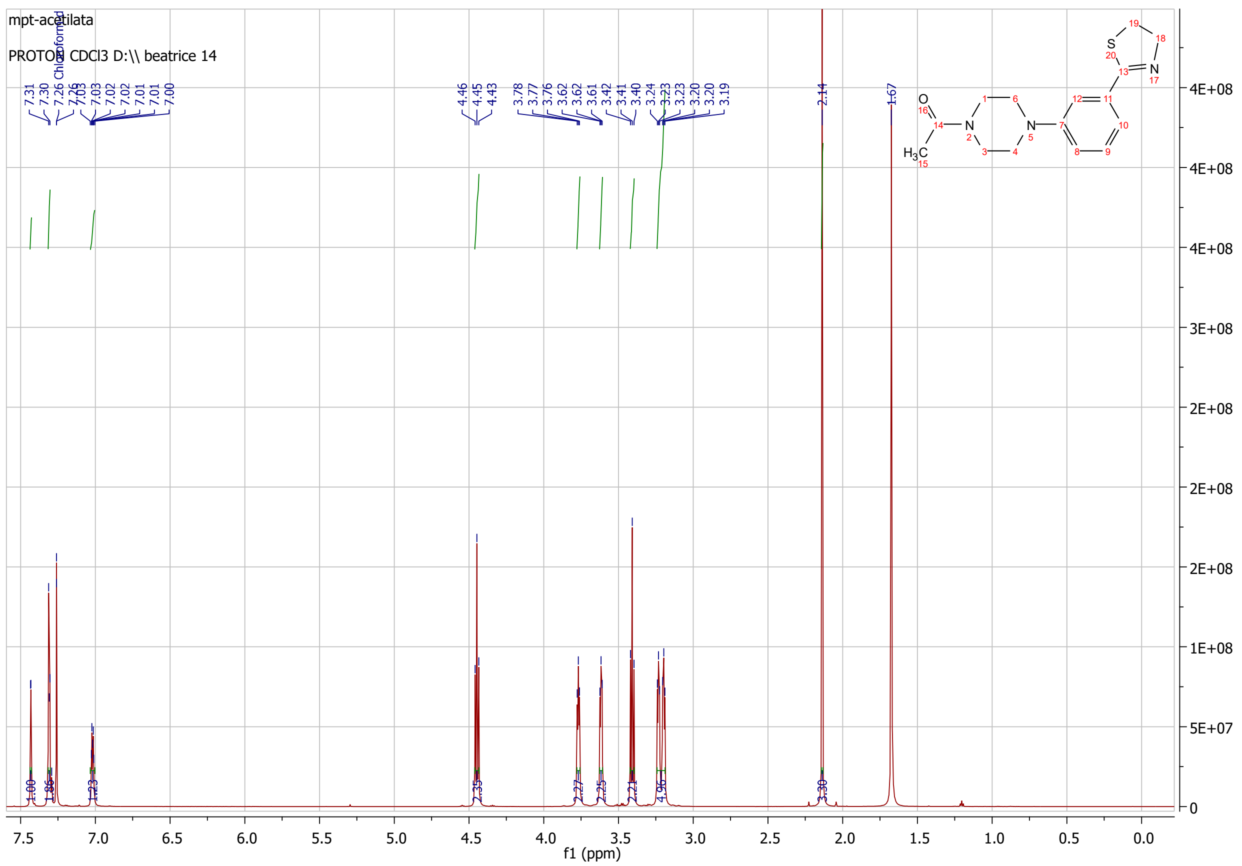
mpt-acetilata
PROTON CDCl3 D:\\ beatrice 14
f1 (ppm)

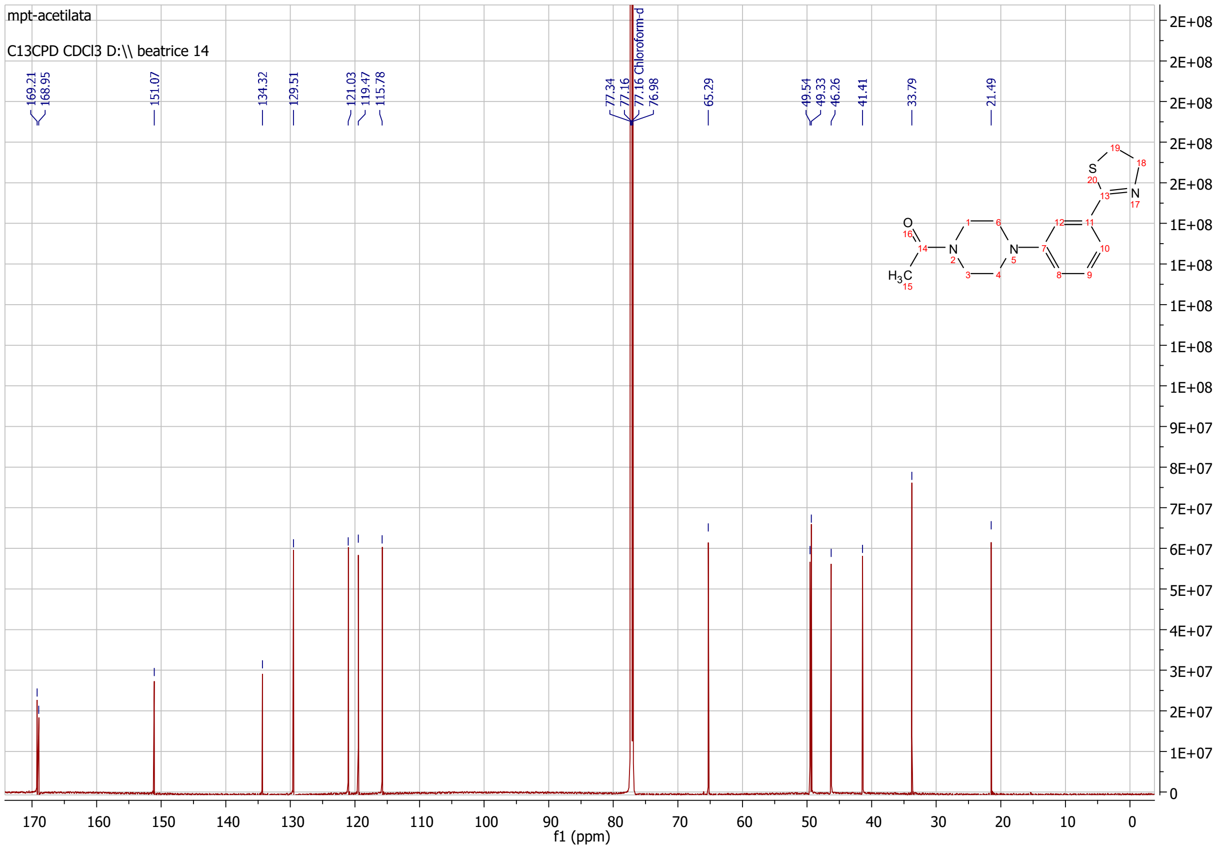
mpt-acetilata
C13CPD CDCl3 D:\\ beatrice 14
f1 (ppm)

Compound 3c

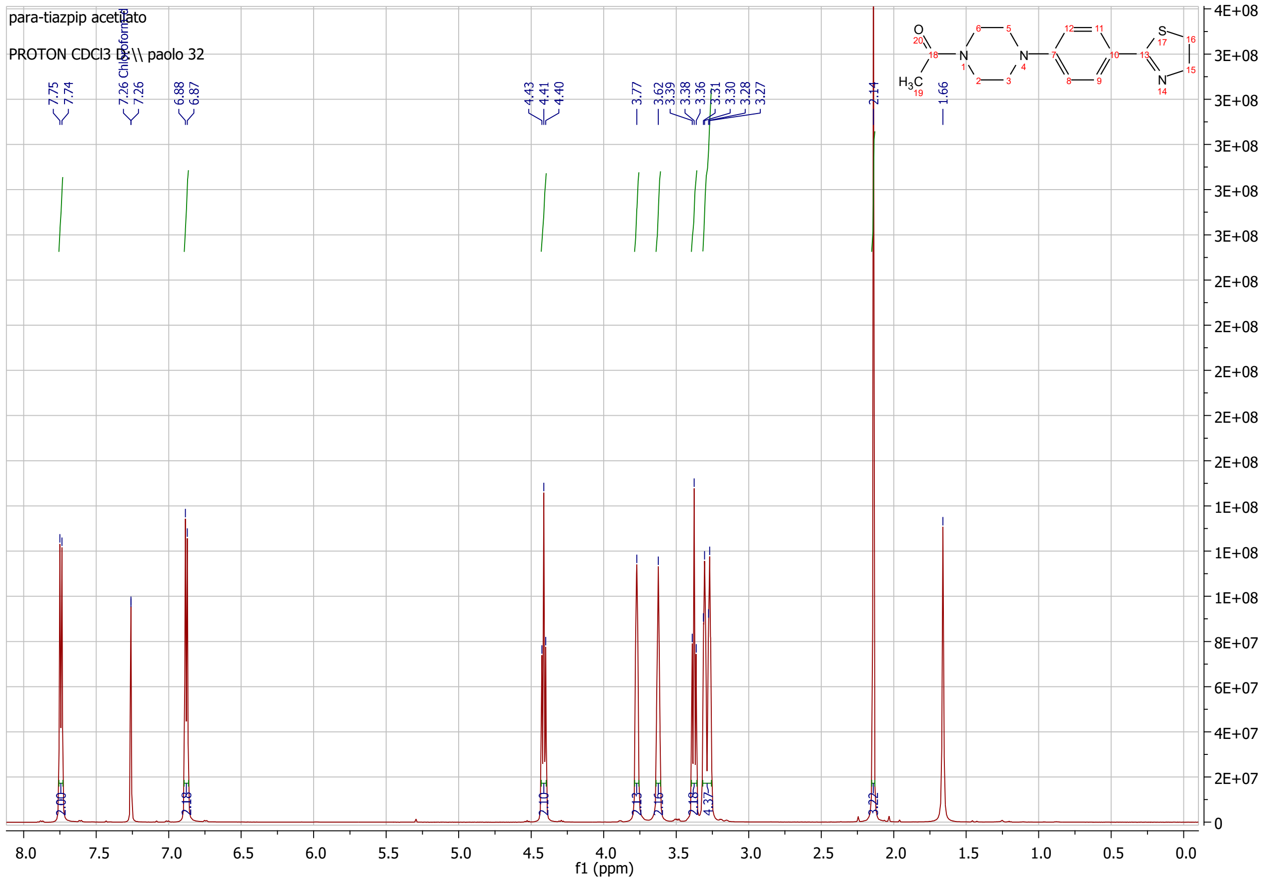
para-tiazpip acetilato
PROTON CDCl3 D:\\ paolo 32
7.75
7.74
7.26 Chloroform-d
7.26
6.88
6.87
4.43
4.41
4.40
3.77
3.62
3.39
3.38
3.36
3.31
3.30
3.28
3.27
2.14
1.66
2.00
2.18
2.10
2.13
2.16
2.18
4.37
3.22
f1 (ppm)

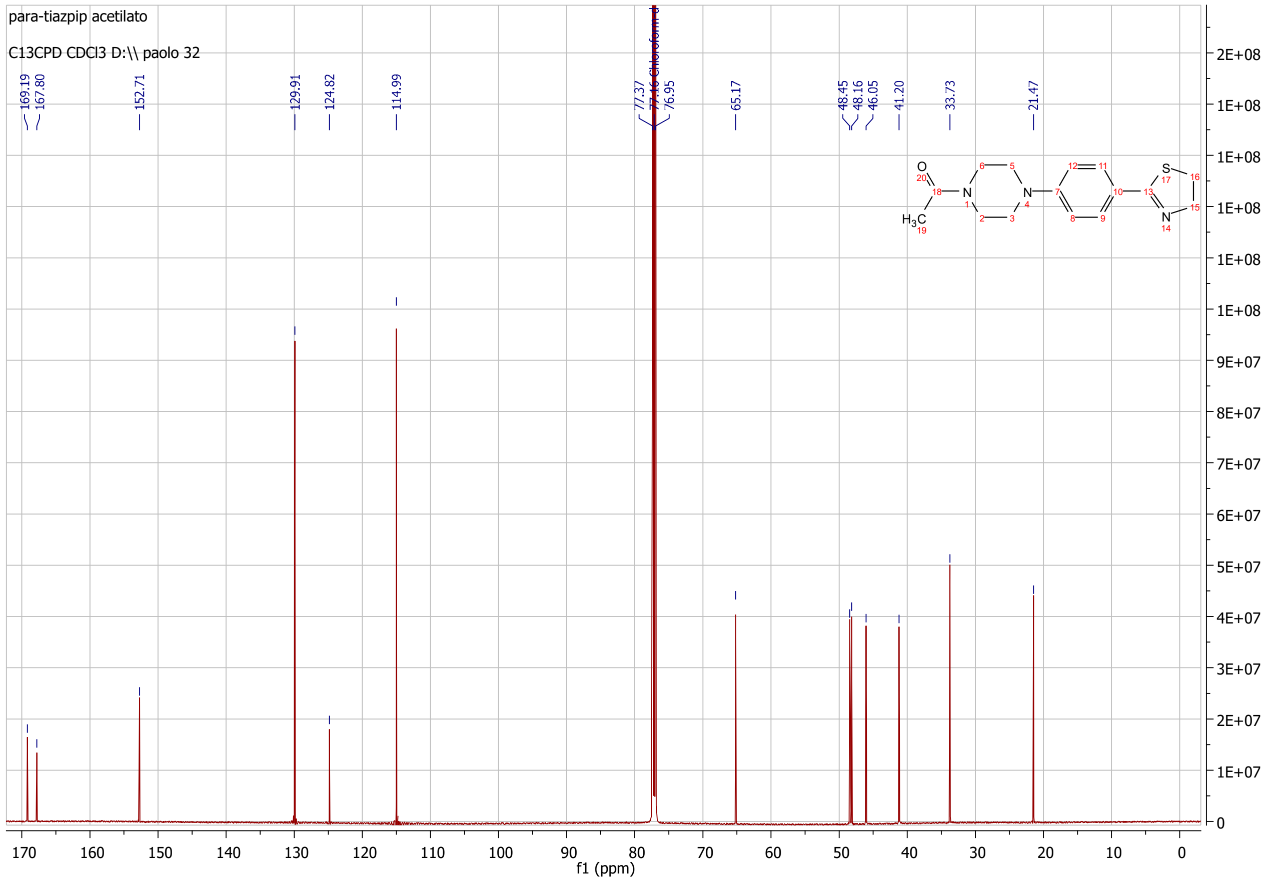
para-tiazpip acetilato
C13CPD CDCl3 D:\\ paolo 32
169.19
167.80
152.71
129.91
124.82
114.99
77.37
77.16 Chloroform-d
76.95
65.17
48.45
48.16
46.05
41.20
33.73
21.47
f1 (ppm)
